# Chronic Kidney Disease in Balkan Countries—A Call for Optimal Multidisciplinary Management

**DOI:** 10.3390/ijerph22020140

**Published:** 2025-01-22

**Authors:** Mario Laganović, Radomir Naumović, Milena Nikolova, Petar Petrov, Josipa Radić, Igor Mitić, Andreja Marn Pernat

**Affiliations:** 1 Department of Nephrology, Clinical Hospital Merkur, 10000 Zagreb, Croatia; 2 Clinic of Nephrology, Zvezdara University Clinical Hospital, 11000 Belgrade, Serbia; radomirnaumovic450@gmail.com; 3 Clinic of Nephrology, University Hospital “St Ivan Rilski”, 1000 Sofia, Bulgaria; milena_i_dani@abv.bg; 4 Clinic of Nephrology, University Hospital St. Marina, 9010 Varna, Bulgaria; petar.petrov@mu-varna.bg; 5 Department of Nephrology, University Hospital Centre Split, School of Medicine, University of Split, 21000 Split, Croatia; jradic@kbsplit.hr; 6 Clinic for Nephrology and Clinical Immunology, University Clinical Center of Vojvodina, 21000 Novi Sad, Serbia; igor.mitic@mf.uns.ac.rs; 7 Medical Faculty, University of Ljubljana, University Medical Center Ljubljana, 1000 Ljubljana, Slovenia; andreja.marn@kclj.si

**Keywords:** chronic kidney disease, CKD, treatment, SGLT2 inhibitor, Balkan, Eastern Europe

## Abstract

The treatment of chronic kidney disease (CKD) has been considerably transformed in the last couple of years. However, effective management of patients with CKD is still not achieved, despite clear guidelines promoting active screening of high-risk patients, immediate diagnosis based on laboratory markers, and early initiation or intensification of pharmacotherapy like sodium/glucose cotransporter 2 (SGLT2) inhibitors, which showed reliable results in preventing disease progression, complications, and mortality. Following a recent initiative on early diagnosis, nephrology experts from Bulgaria, Croatia, Serbia, and Slovenia discussed the challenges and opportunities related to CKD treatment in the Balkan countries, also reflecting on the heterogenous socio-economic context of the region. The ongoing education of all stakeholders involved in kidney care, structured support for primary care providers, and the improvement of multidisciplinary networks were consistently recognized as key success factors. Optimal CKD management is based on continuity of care and the timely transition of coordination from primary care to nephrology-specialized services.

## 1. Introduction

Chronic kidney disease (CKD) represents a challenge for healthcare systems due to the significant mortality and morbidity across the stages of the disease, as well as the substantial economic burden. Despite increasing efforts to promote disease awareness globally and in Europe, most people with laboratory results-based CKD do not have this diagnosis documented and potentially lack an optimal management strategy [1].

With a relatively easy diagnosis and clear treatment algorithm [2,3,4], CKD is still considered a complex disease for various reasons. Symptoms are not specific and may be absent or mild until advanced stages, and kidney damage is often a comorbidity or complication of other conditions, like obesity, diabetes, and cardiovascular disorders. The patient’s journey is complicated by multiple encounters with different healthcare professionals (HCPs), often in decentralized healthcare systems with inefficient communication lines between providers. Inappropriate or duplicate testing and delayed treatment have significant adverse consequences for these patients [5,6].

In the management of CKD, primary care physicians (PCPs) and nephrologists are the critical pillars [6,7]. The leading role may change according to the stage of the disease and occurrence of complications, with PCPs having the key involvement in the prevention, screening, and initiation of treatment in the early stage, and nephrologists taking the lead when advanced interventions are needed. These specialists share responsibility in the first place to reduce the risk of mortality, progression of kidney disease, and development of cardiovascular, renal, and metabolic complications in people with CKD. Other healthcare professionals involved in the management of patients with CKD, such as cardiologists and diabetologists, are also important, considering the opportunity to discover and manage CKD in the early stages. Frequently, focusing on their own treating condition, these specialists are not sufficiently familiar with the comorbidities and complications of CKD, which becomes particularly important in the era of new drugs that can significantly slow down the progression of CKD and improve the prognosis of these patients [8].

Results achieved with new medicines to improve outcomes in patients with established CKD [9,10] have led to effervescent communication around the “neglected kidney condition” [11], updates of clinical guidelines [2,3,4], and perspectives of better outcomes. For the first time in many years, a significant reduction in mortality rates has been shown, improving the patients’ health perspectives [9]. Improvements in kidney care and opportunities for implementing guideline-based treatment algorithms have been noticed in Central and Eastern Europe, although the region lags behind Western countries from an economic perspective [12,13]. Inequalities in healthcare across European countries, corroborated by generally low rates of national income, treatment rates, number of healthcare specialists per million people, and inadequate research in CKD, increase challenges in kidney care [13].

CKD represents a significant problem in our region. Although robust epidemiological data are not available, estimates on the prevalence of CKD are 10–12%, as we showed by analyses performed at the international level and included in our previous publications [14,15].

Related to the call for action for screening and diagnosis of CKD [14], a panel of eight nephrologists from Bulgaria, Croatia, Serbia, and Slovenia discussed the realities and actions needed in the Balkan region for early optimal CKD management. Like the previous meeting, the experts presented the current status of treatment of patients with CKD in their clinics, referral pathways, and steps taken in education and improvement of collaboration. The opinions presented below were unanimously agreed upon by the participants. This manuscript provides perspectives on achieving optimal kidney care in the region and emphasizes the need for optimal interdisciplinary management.

## 2. Assessment of CKD Stage and Early Initiation of Treatment

The definition of CKD by the Kidney Disease—Improving Global Outcomes (KDIGO) guidelines is currently based on the presence of an estimated glomerular filtration rate (eGFR) < 60 mL/min/1.73 m^2^ and/or a urinary albumin- or protein-to-creatinine ratio (uACR ≥ 30 mg/g or uPCR ≥ 150 mg/g) and/or markers of kidney disease present for at least 3 months [2,3]. Based on the eGFR, five stages of the disease have been identified, from G1 (eGFR ≥ 90 mL/min/1.73 m^2^, considered normal or high) to G5 (eGFR < 15 mL/min/1.73 m^2^, termed kidney failure) [2]. It is important to note that the CKD diagnosis may be present even with high eGFR values, pointing to the need for thorough assessment in patients at risk [16].

**Figure 1:**
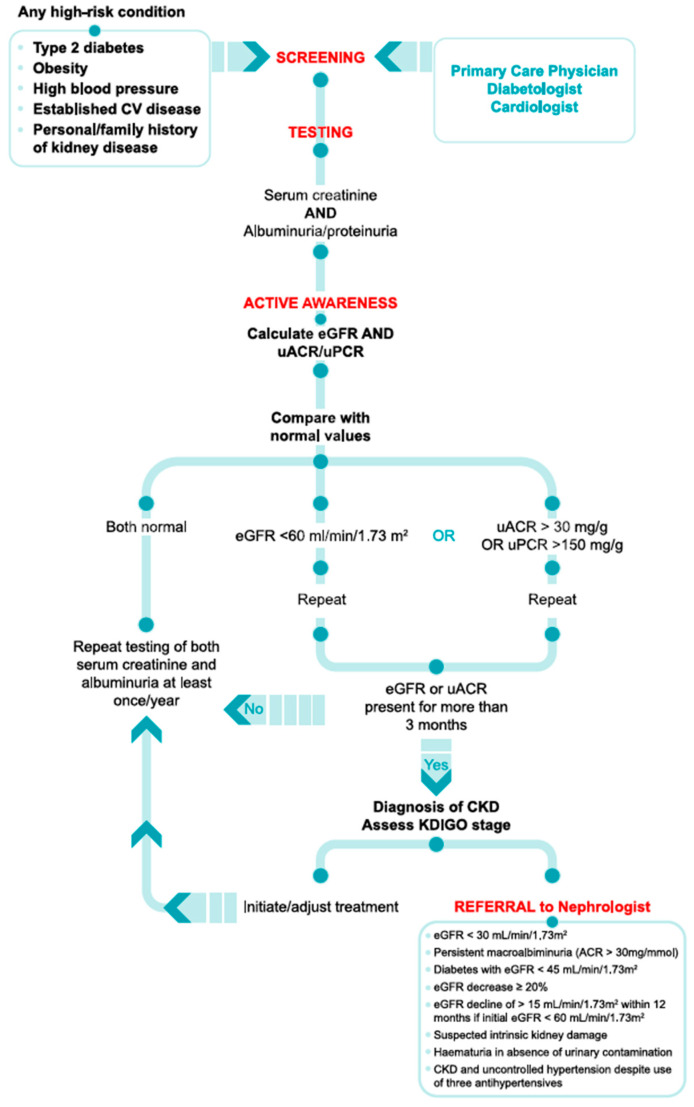
Suggested algorithm for screening, diagnosis, monitoring, and referral of patients with CKD in clinical practice. Abbreviations: CV, cardiovascular; CKD, chronic kidney disease; eGFR, estimated glomerular filtration rate; KDIGO, Kidney DiseaseImproving Global Outcomes; uACR, urinary albumin-to-creatinine ratio; uPCR, urinary protein-to-creatinine ratio. Note: diagnosis and monitoring indicated in the algorithm take into account only laboratory values of creatinine (eGFR), and albuminuria or proteinuria (uACR or uPCR). According to KDIGO guidelines [2,3], diagnosis of CKD may also be based on different structural kidney changes identified by biopsy or imagistic exams, urine sediment abnormalities, and/or electrolyte imbalances due to tubular disorders.

PCPs have most of the opportunity to screen the populations considered to be at high risk of CKD (diabetes, arterial hypertension, cardiovascular disorders, obesity, and/or a history of kidney disease), discuss the need for repetitive laboratory testing and the implications of early diagnosis, and take initial therapeutic measures. Optimal management is different in the pre-diagnosis phase (identification of persons to be screened for CKD, obtaining their commitment for laboratory analysis), diagnosis (correct staging based on KDIGO matrix) [2,3], and early treatment (control of modifiable risk factors and initiating the first-line treatment). Therefore, the first crucial step is that PCPs become aware and actively involved in the process, and we call this “active awareness” (Figure 1).

## 3. Opportunities and Challenges

### 3.1. Laboratory Testing

Testing of serum creatinine and albuminuria/proteinuria is fully reimbursed in primary care in Bulgaria, Croatia, and Slovenia. Although the eGFR value may be automatically calculated and reported by the laboratories, this is not the current practice in the Balkan region, being implemented and functional only in Slovenia and inconstantly implemented in the rest of the countries. If not calculated by the laboratory, the eGFR is determined by the PCP or by another specialist involved in the patient’s care (mainly an endocrinologist, nephrologist, or cardiologist), usually using Modification of Diet in Renal Disease (MDRD) or Chronic Kidney Disease Epidemiology Collaboration (CKD-EPI) equation. An optimal equation to obtain the eGFR in obese patients is still under debate, with potential solutions arising to avoid misdiagnosis [17].

Nevertheless, implementation of a software adjustment to include in the laboratory analysis report both the eGFR and serum creatinine may support the early diagnosis process by raising an alarm signal for the treating physician, and we consider it to be a minimum requirement as part of the optimal management strategy. This technical update was implemented in the US almost 20 years ago; however, its true contribution to the improvement in CKD diagnoses is uncertain [18]. CKD diagnosis and monitoring is almost entirely based on laboratory measurements, so the automatization of the process would potentially improve the odds and transform a lost opportunity into a window of opportunity for our patients. Newer biomarkers of kidney damage could also be considered, e.g., Neutrophil Gelatinase-Associated Lipocalin (NGAL), Kidney Injury Molecule-1 (KIM-1), Beta-Trace Protein (BTP), Beta-2 Microglobulin (B2M), Uromodulin, Asymmetric Dimethylarginine (ADMA), Symmetric Dimethylarginine (SDMA), Metabolomic and Proteomic Biomarkers, and MicroRNAs (miRNAs), depending on the capabilities of the center itself. Since they are not used in everyday clinical practice in our countries, we have not evaluated in more detail their potential role in the early diagnosis of CKD.

### 3.2. Patients’ Education

Surveys of patients with CKD show that they are not generally informed about having this diagnosis [19,20]. Even after the communication of the diagnosis, a proportion of patients do not understand their health problem and do not accept it. A pilot educational program for patients in Croatia offers an online tool to calculate their eGRF and brief explanations in Croatian on the stage of kidney disease, treatment options, as well as dietary recommendations according to their kidney function [21]. The program is also extended for PCPs, with tailored information to support early diagnosis of CKD and optimal monitoring through electronic health records.

A considerable amount of information is easily available on the internet, and it might be difficult to filter the incorrect or irrelevant information. A survey of patients with dialysis showed they trust their medical teams and some of them value meeting and talking with other patients with the same condition [22]. In practice, informative materials, including healthy meal plans, developed in Bulgaria (by nephrologists and dietitians), Croatia (by the Society for Nephrology, Dialysis and Transplantation), and Slovenia (by the Nephrology Society), were appreciated by patients and their families. Educational needs might be different at the time of disease progression, from general changes in lifestyle and avoidance of risk factors to the role of first-line treatment, then preparation for dialysis and specific therapeutic measures. Tailoring communication to patients’ needs in everyday practice translates into the provision of consistent and reliable information and the maintenance of open communication.

### 3.3. Front-Line Medical Staff

As with many other chronic diseases, the majority of patients with CKD can be managed in primary care. Nevertheless, without adequate training, the CKD diagnosis may be overlooked. Recent data show that almost half of the patients with laboratory values indicative of CKD did not have a documented diagnosis in their health records [18,23].

In Balkan countries, the concept of primary care in CKD has extended from family medicine to specialists in diabetes/endocrinology and even cardiologists and nephrologists, representing the first significant decision level in kidney care. The primary care team usually consists of a physician and a nurse. A nutritionist and/or diabetes educator is usually included in the network, mostly in cases of patients with type 2 diabetes. Moreover, these HCPs are responsible for education and communication on general lifestyle measures (physical activity, smoking cessation, weight control, reduction of high blood pressure and glucose). It is unrealistic to think of PCPs as being aware and knowledgeable about guidelines and treatment recommendations for all chronic diseases. Therefore, to achieve our aim of optimal management in early CKD, we strongly support activities providing the minimum required tailored information for PCPs, both as web-based educational tools and in the form of direct communication.

Screening of CKD in patients with type 2 diabetes, obesity, or high blood pressure is slowly adopted in routine practice. Basically, all patients with identified risk factors have a “suspicion” of CKD, even in the absence of symptoms or complaints. An ample initiative of screening of over 400 high-risk patients in Croatia, as part of the “Plan for early CKD identification”, showed that 23% of patients had CKD based on laboratory findings; however, they did not have this diagnosis recorded and were not aware of their kidney problem [24]. The program is ongoing and will include an electronic educational tool for patients with low eGFR values and/or increased albuminuria, in addition to other informative sections dedicated to HCPs. Although not directly related to primary care, random testing in a nephrology clinic in Bulgaria led to an increase in CKD diagnoses of 39% [25].

In patients with a documented diagnosis of CKD, family doctors are currently allowed to initiate treatment with renin–angiotensin–aldosterone system inhibitor (RAASi), and these medications are fully reimbursed in all Balkan countries. Moreover, they continue the prescription with a sodium/glucose cotransporter 2 inhibitor (SGLT2i) if the treatment was initiated by a nephrologist, diabetologist, or cardiologist. In addition, the Slovenian model, with full reimbursement of the SGLT2i (dapagliflozin and empagliflozin) in CKD in primary care, supports optimal management in the early stages. Regular monitoring of renal laboratory parameters is important to evaluate the efficiency of therapeutic measures and detect the conditions requiring timely referral to a nephrologist [Figure 1].

## 4. Call for Early CKD Management in Primary Care

### 4.1. Educational Needs

#### 4.1.1. For Patients

- General measures to reduce cardiovascular and renal risk—structured support for lifestyle changes, caution in recommendation of nephrotoxic drugs;
- Dietary-specific information correlated with the CKD stage, developed in collaboration with certified nutritionists;
- Printed or electronic educational materials and tools (mobile applications) regarding kidney health and the importance of simple laboratory monitoring and adherence to treatment.

#### 4.1.2. For Primary Care Staff

- Promotion of screening practices—assessment of both serum creatinine and albuminuria/proteinuria at least once per year, simple instructions about the formula for the eGFR, and KDIGO diagnostic criteria and staging matrix;
- Provision of tools (“How to” models) for communication with patients regarding the diagnosis, the importance of regular monitoring, and initiation of therapeutic measures.

#### 4.1.3. For Allied Professions (e.g., Cardiologist, Endocrinologist)

- Education through joint scientific meetings.

### 4.2. Resources

- Implementation of automatic reporting of the eGFR together with the serum creatinine in laboratory reports;
- Advocate for full reimbursement for diagnostic testing, and availability of results to patients and treating physicians;
- Provide printed materials, including regarding CKD screening, in ongoing diabetes/obesity/cardiovascular programs:
- Create alerts in electronic systems already available in medical offices to signal the presence of high-risk patients and the required frequency of testing;
- Update prescribing protocols by offering PCPs the possibility to initiate the treatment with first-line medications.

## 5. Referral to Nephrologist

KDIGO recommendations for referral to a nephrologist were recently updated [3]. In addition to patients requiring advanced measures of kidney replacement therapy, other categories of patients with early CKD may benefit from specialized nephrology services. The risk of severe complications would be reduced in patients at risk, while unnecessary visits in secondary care might be avoided. If referral recommendations would be based on the estimated 2-year or 5-year progression risk, as suggested by several groups of international experts [26,27], they should be balanced with the overall low number of nephrologists and limited staff availability in secondary or tertiary clinics [28].

As outlined during the meeting, most patients are referred to nephrologists by PCPs, followed by endocrinologists/diabetologists, and cardiologists. In the clinical practice of the participants, a large proportion of patients arriving in nephrology services do not receive the right first-line strategy and are not adequately managed until serum creatinine reaches values above the normal limits and renal function is at least partially lost. For patients with CKD, referral to a nephrologist should not be the moment of treatment initiation. The early use of medication (RAASi plus SGLT2i) is crucial to delay progression, complications, and death. Specialists involved in the treatment of cardiac or metabolic disorders may step in to initiate the prescription of novel therapies with proven benefits to kidney disease, since the reimbursement conditions for SGLT2i treatment are different across countries and indications (Table 1). We expect that current efforts to improve education and timely initiation of treatment prior to referral, as well as the establishment of collaborative networks among providers, will show results in the coming years.

Continuity of care is another major pillar of the optimal treatment pathway, along with diagnosis and treatment, to potentially avoid the occurrence of significant cardiovascular and renal complications, premature death, and time to dialysis or other renal replacement therapies. Discontinuity of kidney care may be related to many factors, from patients’ attitudes and abilities to manage their disease to inconsistency in medical advice and flaws of the healthcare system. Gaps in the management of CKD, even in the early stages, have been associated with higher rates of hospitalization and emergency care visits [28].

## 6. Call for Optimal Time of Referral to Nephrologist

- Promote implementation of electronic health records and automatization processes (e.g., laboratory reports to be available for the multidisciplinary team (MDT), alerts set up for periodic monitoring and specialist visits);
- Advocate for the continuity of CKD care in all meetings organized by nephrology societies and create alliances and partnerships with PCP, cardiology, and diabetes/endocrine professional groups to define the responsibility for diagnosis, referral, and monitoring such that it is integrated into normal clinical practice, and continuity of kidney care:
  ○ Working protocols to bring structure and clarity, not an additional administrative burden, for the medical staff;
  ○ Recognition of the additional time needed for coordinated activities for specialized nurses/patient navigators;
  ○ Templates for referral and back referral letters;
- Continue educational programs for nephrologists (adoption of treatment guidelines, communication with patients, multidisciplinary care).

The coordinated care approach to CKD management is necessary to deploy best practices in chronic disease management that engages the MDT. The backbone of the MDT should include at least the primary care professionals (physician, nurse, dietician) and the nephrologist. The expanded team would welcome the cardiologist, endocrinologist/diabetologist, as well as a mental health professional. Ideally, PCPs should coordinate the care until referral; then, the management of CKD would be led by the nephrologist.

## 7. Discussion on the Unmet Need of Timely Diagnosis and Optimal Treatment of Patients with CKD in the Region

Balkan countries share many cultural characteristics, along with the powerful socio-economic heritage of the presence of one single-party authority in the communist period. Despite the existence of democratic political governments in the last 30 years and the important progress of the nations, regional development still lags behind Western Europe, with significant implications for the decision-making process in the healthcare system [29,30]. The EU recommendations on the approach to CKD emphasize early detection and diagnosis, fast and equitable access to care, including investigations and treatment, and integration of CKD with public health goals, based on standardized recommendations, like KDIGO guidelines, to improve outcomes [31].

Expert nephrologists from Bulgaria, Croatia, Serbia, and Slovenia devoted substantial time to considering productive steps for optimal CKD care in the context of a regional approach adapted to individual healthcare systems. Following the call to action for improvement in the diagnosis of CKD [14], the current paper is centered on strategies for timely and adequate treatment initiation and monitoring to reduce mortality and cardiovascular complications.

Identification of kidney disease is crucial for further management strategy improvements. Currently, diagnosis is based on the current KDIGO guidelines, which represent a major step forward in the management of CKD and have helped with a structured approach in various clinical situations. From a nephrology clinic perspective, the diagnosis of CKD is probably one of the easiest decisions to make in chronic diseases: only two markers are needed, with certain values, confirmed at 3-month intervals. In primary care, where most high-risk patients are seen, establishing the diagnosis may be difficult. In people with confirmed CKD, the estimation of the progression to kidney failure is highly recommended by new KDIGO guidelines [3]. The equation considers the sex and age and eGFR and uACR values, and the geographic location of the patient. As of now, the equation has not been validated in any Balkan country.

Survival messages should be clearly delivered to patients with type 2 diabetes, obesity, and cardiovascular disease, and multiple-layer barriers should be overcome in obtaining their commitment to testing several times per year. Nevertheless, all screening initiatives developed in our countries led to a high proportion of newly diagnosed CKD [24,25]. A strategic approach, at a regional or national level, is urgently required to start or continue the screening programs and monitor the results in the long term. Once the need for the early identification of patients with kidney impairment is acknowledged by decision-makers, solutions to the practical issues of finding resources and continuous education remain to be found.

The SGLT2 inhibitors demonstrated that not only can the risk of kidney disease progression be reduced, but the survival rate can also be improved in this patient population. Although both dapagliflozin and empagliflozin, the two SGLT2 inhibitors available in Europe, showed strong cardio-renal results in people with CKD and different levels of KDIGO risk, only dapagliflozin demonstrated a statistical significance compared with placebo for all-cause mortality risk reduction in patients with CKD [9,10,32]. The first-line treatment with an SGLT2i and RAASi has been strongly recommended in patients with CKD and type 2 diabetes and is promoted by the new KDIGO guidelines, irrespective of diabetes status [3,4]. This strategy has the potential to be rapidly adopted in clinical practice, since the use of these medications has already become the standard of care in diabetes and cardiovascular disease areas. PCPs are in the best position to discuss the role of treatment for life protection and initiate the right medications after diagnosis. Reimbursement for treatment plays a key role in prescribing strategy and adherence. Of all four Balkan countries, the initiation of an SGLT2 inhibitor, irrespective of the indication, has been reimbursed in primary care only in Slovenia. Based on the current experience, no additional burden has been noted, since no supplementary monitoring has been deemed necessary.

A call for optimal CKD management is a call against treatment inertia, translated into an adequate management plan for each patient, from early to advanced stages, considering the practicalities of individual national healthcare systems.

Working with local government bodies in regulating healthcare policies, including national, local, and global screening and educational programs for CKD, is also of great importance to provide early screening and diagnosis and adequate treatment for all CKD patients. Social media platforms can be of great help in the process of the development and spread of information on screening, diagnostic, and educational activities. Future efforts should be aimed at better education of patients, medical professionals, and medical students, and at improving the accessibility of CKD screening and diagnostic tests, and CKD treatment.

## 8. Conclusions

Despite a clear strategy related to CKD management in the Balkans being lacking, educational activities are initiated, and experts take advantage of all opportunities to promote awareness of kidney care as well as improve collaboration through joint research initiatives, data integration and sharing, and conducting comprehensive studies to understand the characteristics of CKD in the region.

In the new treatment paradigm supported by the recently published KDIGO guidelines [3], the focus is more and more on activities performed in primary care. Efforts are needed to continue to build a solid educational base for screening, diagnosis, and optimal treatment, irrespective of the stage at diagnosis, with clear triggers for referral to a nephrology center during disease progression.

In an optimal CKD management journey, the spotlight changes from PCPs in the early stages to nephrologists in the advanced stages. Other specialists treating comorbidities are needed all along the continuum of care, contributing to the personalization of the treatment approach. Regular, frequent, and constant support from nephrologists has been acknowledged as mandatory in the educational endeavor of the multidisciplinary team. With all the medical progress, CKD management would become less difficult, while the patient with CKD would become more complex. Although this review is intended to address the actual situation of CKD in Balkan countries, most of the proposed recommendations are universal and could be applied globally, depending on the capabilities of the center itself or the country in which it is carried out.

## Figures and Tables

**Table 1:** Reimbursement conditions of SGLT2 inhibitors per indication and country (as of September 2024).

Indication	Chronic Kidney Disease (% Reimbursement)	Type 2 Diabetes (% Reimbursement)	Heart Failure (% Reimbursement)	
SGLT2i May Be Prescribed in	Nephrologist	Endocrinologist or Diabetologist	Cardiologist	
			HFrEF	HFpEF
Bulgaria	75%	100%	75%	75%
Croatia	57%	57%	57%	57%
Serbia	NR	100% *	NR	NR
Slovenia	100%	100%	100%	100%

* One hundred percent reimbursement in patients with BMI > 27 kg/m^2^ and poor glycemic control (HbA1c > 7%) despite the presence of at least two previous antidiabetic agents. Note: prescription of an SGLT2i by PCPs is fully reimbursed (100%) for any indication in Slovenia. Prescription of an SGLT2i by PCPs is partially reimbursed (57%) with T2DM indication in Croatia. Abbreviations: HbA1c, glycated hemoglobin; HFpEF, heart failure with preserved ejection fraction; HFrEF, heart failure with reduced ejection fraction; NR, not reimbursed; PCP, primary care physician; SGLT2i, sodium/glucose cotransporter 2 inhibitor.

## Data Availability

All data presented in the manuscript are derived from the published literature indicated in the reference list.
